# Association of Vitamin D Status and Physical Activity with Lipid Profile in Korean Children and Adolescents: A Population-Based Study

**DOI:** 10.3390/children7110241

**Published:** 2020-11-19

**Authors:** Kyungchul Song, Gihong Park, Youngha Choi, Jun Suk Oh, Han Saem Choi, Junghwan Suh, Ahreum Kwon, Ho-Seong Kim, Hyun Wook Chae

**Affiliations:** Department of Pediatrics, Severance Children’s Hospital, Endocrine Research Institute, Yonsei University College of Medicine, Seoul 03722, Korea; endosong@yuhs.ac (K.S.); onenewman@yuhs.ac (G.P.); youngha@yuhs.ac (Y.C.); joojang87@yuhs.ac (J.S.O.); hansaem6890@yuhs.ac (H.S.C.); suh30507@yuhs.ac (J.S.); armea@yuhs.ac (A.K.); kimho@yuhs.ac (H.-S.K.)

**Keywords:** Vitamin D, lipid, physical activity, children, adolescents

## Abstract

Dyslipidemia is one of the important influencing factors of cardiovascular health in the youth, and thus, assessment of its etiology is important. We aimed to investigate the association of dyslipidemia with vitamin D and physical activity in Korean children and adolescents. Data of 3183 subjects aged 12–18 years in the Korea National Health and Nutrition Examination Survey were analyzed. Participants were divided into subgroups according to sex, body mass index, 25-hydroxyvitamin D levels, and lipid profile. The mean 25-hydroxyvitamin D level was 16.15 ng/mL, which was below normal. In total, 79.3% of the subjects had vitamin D deficiency. Females had lower vitamin D levels and a higher incidence of dyslipidemia compared to males. Vitamin D deficiency was significantly associated with high density lipoprotein cholesterol (HDL-C) levels. The low HDL-C group consisted of a higher proportion of subjects with vitamin D deficiency and low physical activity. This study suggests that vitamin D deficiency is prevalent in Korean children and adolescents. Vitamin D deficiency and low physical activity are related with low HDL-C levels. Maintaining sufficient vitamin D levels and physical activity may help prevent dyslipidemia.

## 1. Introduction

Vitamin D is an essential prohormone for the regulation of calcium and bone metabolism [1]. The expression of up to one-third of the human genome is influenced by 1,25-dihydroxyvitamin D, an active form of vitamin D [2]. Furthermore, most tissues and cells in the body have a 1,25-dihydroxyvitamin D receptor. Thus, vitamin D deficiency is related to various diseases including autoimmune and cardiovascular diseases and cancers [2,3]. In addition, vitamin D deficiency has been suggested as one of the possible risk factors of dyslipidemia [1].

Dyslipidemia, characterized by abnormal lipid levels, is a modifiable risk factor for cardiovascular disease, which is the most common cause of death worldwide, accounting for 32% of global deaths [4]. Pediatric dyslipidemia is related to adult dyslipidemia and atherosclerosis as well as childhood atherosclerosis [5]. In this context, the American Heart Association considers dyslipidemia as an important influencing factor of cardiovascular health in youth [6]. In Korea, pediatric dyslipidemia has a prevalence of 19.7% among the general population, similar to the United States (19–25%) [5,7]. Therefore, it is important to assess the etiology and pathophysiology of dyslipidemia in the youth.

Pediatric dyslipidemia is initially managed with lifestyle modification, and medical treatment is considered as second-line management. There are several factors associated with dyslipidemia, including obesity, low physical activity, alcohol consumption, smoking, and diet [8,9,10,11,12]. Among them, physical activity is negatively associated with triglycerides (TG) and positively associated with high-density lipoprotein cholesterol (HDL-C) [13]. HDL-C, an essential cholesterol which maintains cholesterol homeostasis between the artery and organs, is negatively associated with the occurrence of coronary heart disease [14,15]. TG, fatty acid esters of glycerol that are transported in plasma within lipoproteins, are a strong predictor of cardiovascular disease and represent the main lipid component of dietary fat [14,16].

Although vitamin D status has been reported to have a favorable effect on the lipid levels of children and adolescents [17,18,19], no consistent association has been found [20]. Birken et al. [17] reported that 25-hydroxyvitamin D (25OHD) levels are negatively associated with total cholesterol (TC), TG, and non-HDL-C levels in children. Kim et al. [18] reported that subjects with vitamin D deficiency had higher TG levels and TG/HDL-C ratio than subjects with normal vitamin D levels among non-obese children. However, a systematic review reported that no consistent association exists between 25OHD level and lipid levels in children [20].

Thus, this study aimed to investigate the association of lipid levels with vitamin D status and physical activity in adolescents through an analysis of the Korea National Health and Nutrition Examination Survey (KNHANES) data. We hypothesized that subjects with vitamin D deficiency might have higher proportion of dyslipidemia and lower physical activity than subjects with vitamin D sufficiency.

## 2. Materials and Methods

### 2.1. Study Population

This study was approved by the Institutional Review Board of Yonsei University Gangnam Severance Hospital (IRB, 3-2020-0023). All participants volunteered and provided written informed consent prior to study participation.

**Figure 1:**
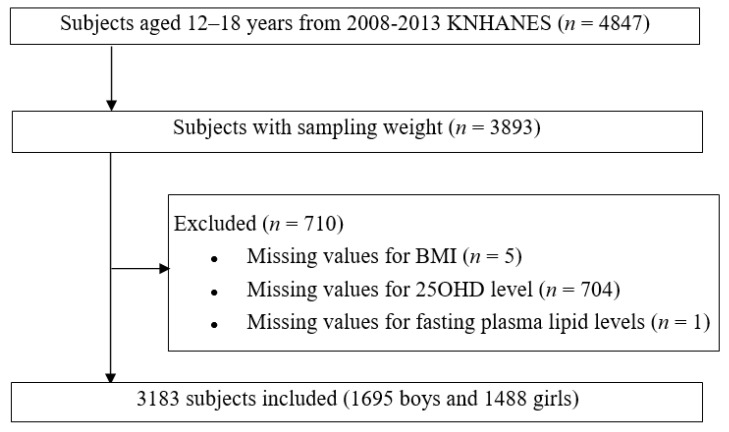
Diagram showing the workflow of the study. KNHANES: Korean National Health and Nutrition Examination Survey; BMI: body mass index; 25OHD: 25-hydroxyvitamin D.

We evaluated 3183 children and adolescents aged 12–18 years who participated in the fourth, fifth, and sixth KNHANES conducted between 2008 and 2013. The KNHANES is a national survey with a stratified and multistage sampling design conducted by the Korea Centers for Disease Control and Prevention based on the National Health Promotion [21]. The overall survey was executed through health interviews, health examinations, and nutrition surveys. The sample weights were constructed for sample participants to represent the Korean population by accounting for the complex survey design and post-stratification. The weights based on the inverses of selection probabilities and response rates were modified by adjusting them to the sex- and age-specific Korean populations. The study design and diagram showing the workflow of the study are shown in Figure 1.

### 2.2. Study Variables

Data on age, sex, anthropometric measurements, physical activity, 25OHD levels, and plasma lipid levels were collected. Height and weight were measured using standard protocols, and body mass index (BMI) was calculated as the weight divided by the height squared. Height and BMI were presented as standard deviation scores and classified based on the 2017 Korean National Growth Charts [22]. Vitamin D status was determined according to 25OHD levels and classified as sufficient and deficient at levels of > 20 ng/mL and ≤ 20 ng/mL, respectively [2,3]. The subjects were asked about the intensity and duration of physical activity during the week. Regular physical activity was defined as moderate or vigorous physical activity for at least 1 hour/day 7 days/week based on the World Health Organization’s recommendations [23].

### 2.3. Measurement of Serum 25OHD and Lipid Profiles

Following an 8-hour fast, blood samples were collected from the antecubital vein, processed, and immediately refrigerated. Serum 25OHD levels were measured using radioimmunoassay (DiaSorin, Stillwater, MN). The coefficient of variation of the control material was < 7.8% and < 5.8% for low and high levels, respectively.

The serum levels of the total cholesterol (TC), HDL-C, and TG were measured using a Hitachi Automatic Analyzer 7600/7600-210 (Hitachi, Tokyo, Japan). Low-density lipoprotein cholesterol (LDL-C) was calculated using the Friedewald formula (LDL-C = TC − [HDL-C + (TG / 5)]) [24]; non-HDL-C was calculated as TC − HDL-C [25]. Dyslipidemia was defined using one of the following cutoffs specified in the American Academic of Pediatrics guidelines [11]: TC ≥ 200 mg/dL; LDL-C ≥ 130 mg/dL; TG ≥ 130 mg/dL; HDL-C < 40 mg/dL; or non-HDL-C ≥ 145 mg/dL.

### 2.4. Statistical Analysis

Sampling weights were considered in all analyses to report representative estimates of the Korean adolescent population. All continuous variables were expressed as weighted means with standard errors, whereas categorical variables were expressed as weighted percentages and standard errors. We divided the subjects into subgroups according to sex, BMI percentile, 25OHD levels, and lipid level values, and subgroups analyses were performed using Student’s *t*-test to analyze the mean differences among the subgroups. The Rao-Scott Chi-square test was used to analyze the association of categorical variables with dyslipidemia and vitamin D deficiency groups. All statistical analyses were performed using SAS version 9.4 (SAS Institute, Cary, NC, USA) for the complex survey design with clustering, stratification, and unequal weighting of the KNHANES sample. A *p*-value of < 0.05 was considered significant.

## 3. Results

### 3.1. Baseline Characteristics of Subjects and Comparisons According to Sex

**Figure 2:**
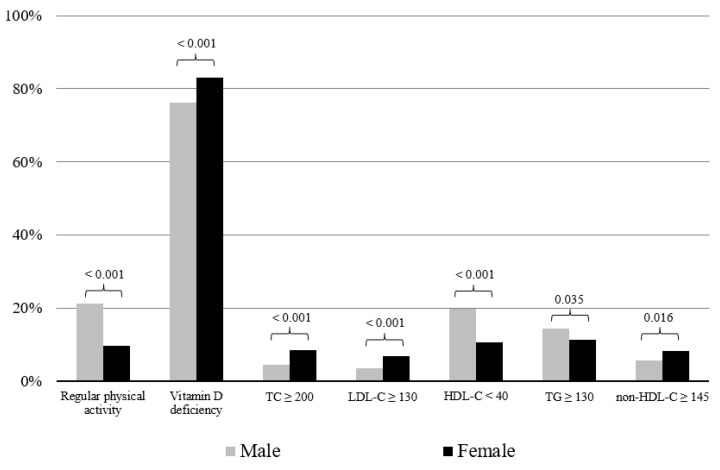
Percentage of subjects with regular physical activity, vitamin D deficiency, and dyslipidemia according to sex. The *p*-value of each independent t-test is presented above the bars. Regular physical activity was defined as moderate or vigorous physical activity for at least 1 hour/day 7 days/week. Vitamin D deficiency was defined as 25-hydroxvitamin D ≤ 20 ng/mL. TC: total cholesterol; LDL-C: low-density lipoprotein cholesterol; HDL-C: high-density lipoprotein cholesterol; TG: triglycerides.

Table 1 shows the baseline characteristics according to sex. In total, 79.29% of subjects had vitamin D deficiency. Physical activity and 25OHD levels were higher in males than in females. Mean HDL-C level was lower in males than in females. Figure 2 shows the percentage of subjects with regular physical activity, vitamin D deficiency, and dyslipidemia according to sex. The proportion of subjects with regular physical activity was higher in males than in females, while the proportion of subjects with vitamin D deficiency was higher in females than in males. In addition, the proportion of subjects with dyslipidemia defined as high TC, LDL-C, or non-HDL-C was higher in females than in males, while the proportion of subjects with HDL-C < 40 mg/dL was higher in males than in females.

### 3.2. Association of Dyslipidemia and Physical Activity According to Sex and 25OHD Levels Among the Subjects

Table 2 shows the comparison of the proportion of subjects with dyslipidemia and physical activity in the vitamin D deficiency and sufficiency groups according to sex and BMI. Among boys with overweight and obesity, the vitamin D deficiency group included higher proportion of subjects with HDL-C < 40 mg/dL than the vitamin D sufficiency group. However, discrepant findings were found in girls with normal BMI, overweight, or obesity. In addition, boys with vitamin D deficiency had lower physical activity than those with vitamin D sufficiency.

Table 3 shows the proportion of subjects with vitamin D deficiency and physical activity in the dyslipidemia and normal lipid groups according to sex and BMI. There were no specific significant findings, except HDL-C in the normal BMI group. The subjects with HDL-C < 40 mg/dL included a higher proportion of subjects with vitamin D deficiency than in the normal lipid group among boys with overweight or obesity. However, discrepant findings were found in girls with normal BMI, overweight, or obesity. In addition, boys with high LDL-C or non-HDL-C levels had lower physical activity than the normal group.

## 4. Discussion

The effect of vitamin D status on the lipid levels of children and adolescents has yet to be clarified. Our study found that vitamin D deficiency positively correlated with dyslipidemia defined by HDL-C in boys who were overweight or obese. Girls had lower vitamin D levels and a higher incidence of dyslipidemia compared to boys. In addition, for boys with overweight or obesity, subjects with vitamin D sufficiency had higher physical activity compared to those with vitamin D deficiency, while those with dyslipidemia had lower physical activity than those with normal lipid levels.

Previous studies that investigated the association between vitamin D status and dyslipidemia showed different results. A systematic review and meta-analysis of pediatric studies showed that 25OHD levels were negatively correlated with TC, TG, and LDL-C and positively correlated with HDL-C [19]. However, another systematic review found an inconsistent association between 25OHD and lipid levels in children [20]. Rusconi et al. [26] found that 25OHD levels were negatively associated with TC levels in obese children. Kim et al. [18] showed that 25OHD levels were negatively correlated with TG levels and the TG/HDL ratio in non-obese children.

The mechanism accounting for the positive correlation between 25OHD and HDL-C can be explained by the association between vitamin D and apolipoprotein A-1. 1,25-dihydroxyvitamin D suppresses apolipoprotein A-1 gene expression by altering the activities of coactivators or corepressors [27]. Apolipoprotein A-1 is the main component of HDL-C, and thus, the capability of vitamin D to maintain adequate levels of apolipoprotein A-1 has been suggested as a potential mechanism for the positive correlation between vitamin D with HDL-C in the youth [28]. Auwerx et al. found a positive correlation between 25OHD level and apolipoprotein A-1 level in adults [29]. However, HDL-C levels negatively correlated with 25OHD levels in girls in our study. The difference in HDL-C between boys and girls in response to vitamin D suggests that there may be more reasons than just vitamin D and/or physical activity, including sex differences. Arnberg et al. speculated that the reduction in HDL-C levels resulting from vitamin D supplements may be due to a concordant reduction in cholesterol levels [30]. Our finding of high TC levels in females with vitamin D deficiency supports this previous finding.

The mechanisms by which vitamin D could affect TG and LDL-C are not clear. However, several mechanisms are suggested: (1) The vitamin D receptor is expressed ubiquitously, including in adipose tissue, which determines cholesterol levels through regulating bile acid synthesis from cholesterol [31]. (2) Increasing calcium absorption stimulated by vitamin D and parathyroid hormones may reduce intestinal fatty acid absorption through the formation of insoluble calcium-fatty complexes and reduce the synthesis and secretion of hepatic TG [1]. Decreased fat absorption would reduce LDL-C levels [32]. In addition, calcium could reduce cholesterol levels by promoting the conversion of cholesterol into bile acids [33]. (3) Vitamin D is associated with the modulation of beta cell function and insulin resistance [34].

In this study, the mean 25OHD level was low (16.15 ng/mL) and the proportion of subjects with vitamin D deficiency was high (79.29%). Further subgroup analysis found a higher mean 25OHD level in boys than in girls and a higher prevalence of vitamin D deficiency among girls. Despite the well-known association between vitamin D and various diseases, hypovitaminosis D remains prevalent among youth. A total of 48% of white prepubertal girls and more than 50% of Hispanic and African-American adolescents in the Unites States have vitamin D deficiency [2]. In Korea, approximately 70% of adolescents has vitamin D deficiency [35]. In a study by Schleicher et al., using data from the US National Health and Nutritional Examination Survey, the mean 25OHD level of adolescents was approximately 25 ng/mL [36]. Meanwhile, it was 17.24 ng/mL for boys and 15.68 ng/mL for girls in a KNHANES-based study [35]. The survey season, the amount of skin covered by clothing, and lack of sun exposure might have an important influence on the vitamin D levels in general. Furthermore, the lower vitamin D status in Koreans may be due to lower consumption of vitamin D-fortified foods, such as milk. In contrast with Korea, various vitamin D-fortified foods are popular in western countries due to national policies and programs that encourage vitamin D consumption [35,37].

Consistent with previous findings [35,36], we found a lower 25OHD level in girls than in boys. Moreover, girls showed lower physical activity than boys, and the boys with vitamin D deficiency had lower physical activity than the boys with sufficient vitamin D. These findings suggest that a low vitamin D status in girls may be due to low sun exposure from lower outdoor activities. Thus, sufficient outdoor activities required to maintain optimal vitamin D status. Physical activity is an important component of lifestyle modification for preventing dyslipidemia in youth [13]. LeBlanc et al. [13] reported that moderate-to-vigorous physical activity lowered the risk of low HDL-C levels and high TG levels in youth. In our study, LDL-C and non-HDL-C levels were associated with lower physical activity among boys. Globally, insufficient physical activity, evaluated based on the World Health Organization’s recommendations, is prevalent in 81.0% of adolescents, and the rate is even higher in South Korea (94.2%) [23]. The prevalence of insufficient physical activity in this study was also higher compared to the global statistics. Programs for increasing physical activity are needed to lower the risk of dyslipidemia in adolescents with insufficient physical activity.

In this study, TC, LDL-C, TG, and non-HDL-C levels were lower in boys than in girls. In addition, the proportion of subjects with dyslipidemia with high TC, LDL-C, or non-HDL-C was lower in boys than in girls, while those with low HDL-C were higher in boys than girls. These results were consistent with a previous KNHANES-based study [7,38]. In the United States, boys had lower TC levels compared to girls [38]. Higher lipid levels in girls may be associated with lower physical activity and 25OHD levels compared to those in boys. Further studies are required to examine possible explanations for the higher lipid levels in girls.

This study has some limitations. First, this study was a cross-sectional study and included only Korean individuals, thus limiting the generalizability of our study. Second, although plasma lipid levels are associated with pubertal development [7], we could not consider pubertal status in the analysis because the KNHANES database does not include information regarding the pubertal stage. Third, information regarding confounders of 25OHD levels, such as sun exposure, usage of sunscreen, season, wearing of long sleeves and pants, and vitamin D supplementation were not considered. Fourth, multivariable logistic regression analysis is needed to determine the associations between Vitamin D, physical activity, and dyslipidemia. Despite these limitations, we believe that our study remains valuable because it addresses the lack of research on the association of physical activity with dyslipidemia and vitamin D status. Furthermore, we evaluated a large number of children and adolescents including obese and non-obese individuals.

## 5. Conclusions

This study demonstrated that vitamin D deficiency may be one of the risk factors of dyslipidemia. Sufficient physical activity may lower the risk of vitamin D deficiency and dyslipidemia. Therefore, efforts to maintain sufficient vitamin D status and increase physical activity should be taken to maintain optimal lipid levels in children and adolescents. 

## Figures and Tables

**Table 1:** Baseline characteristics of subjects.

	Total (*n* = 3183)	Male (*n* = 1695)	Female (*n* = 1488)	*p*
Age (year), mean (SE)	15.09 (0.04)	15.11 (0.05)	15.08 (0.06)	0.709
Height (cm), mean (SE)	165.35 (0.20)	169.73 (0.25)	160.06 (0.19)	< 0.001
Weight (kg), mean (SE)	58.03 (0.29)	61.81 (0.40)	53.46 (0.33)	< 0.001
BMI (kg/m^2^), mean (SE)	21.08 (0.08)	21.31 (0.11)	20.82 (0.11)	0.002
BMI SDS, mean (SE)	−0.08 (0.03)	−0.12 (0.04)	−0.03 (0.04)	0.095
BMI group, percentage (SE)				0.712
Normal (%)	80.71 (0.81)	80.10 (1.14)	81.44 (1.26)	
Overweight (%)	9.68 (0.60)	9.84 (0.84)	9.49 (0.88)	
Obesity (%)	9.61 (0.65)	10.06 (0.93)	9.07 (0.98)	
Physical activity (hour/week), mean (SE)	4.10 (0.18)	5.33 (0.27)	2.62 (0.19)	< 0.001
25OHD (ng/mL), mean (SE)	16.15 (0.16)	16.78 (0.20)	15.40 (0.19)	< 0.001
TC (mg/dL), mean (SE)	155.72 (0.66)	150.64 (0.87)	161.88 (0.84)	< 0.001
LDL-C (mg/dL), mean (SE)	88.95 (0.55)	85.57 (0.75)	93.05 (0.74)	< 0.001
HDL-C (mg/dL), mean (SE)	49.75 (0.23)	47.93 (0.27)	51.96 (0.33)	< 0.001
TG (mg/dL), mean (SE)	85.09 (1.19)	85.73 (1.64)	84.32 (1.53)	0.507
Non-HDL-C (mg/dL), mean (SE)	105.97 (0.61)	102.71 (0.84)	109.91 (0.81)	< 0.001

Values are presented as means (standard error), and categorical data are presented as percentages (standard error). Regular physical activity was defined as moderate or vigorous physical activity for at least 1 hour/day 7 days/week. Vitamin D deficiency was defined as 25OHD ≤ 20 ng/mL. SE: standard error; BMI: body mass index; SDS: standard deviation score; 25OHD: 25-hydroxyvitamin D; TC: total cholesterol; LDL-C: low-density lipoprotein cholesterol; HDL-C: high-density lipoprotein cholesterol; TG: triglycerides.

**Table 2:** Proportion of subjects with dyslipidemia and physical activity among the youth according to sex, BMI, and 25OHD levels.

Male (*n* = 1695)	Normal (*n* = 1358)			Overweight or Obesity (*n* = 337)		
	Vitamin D Deficiency (*n* = 993)	Vitamin D Sufficiency (*n* = 365)	*p*	Vitamin D Deficiency (*n* = 133)	Vitamin D Sufficiency (*n* = 41)	*p*
TC ≥ 200, percentage (SE)	2.39 (0.61)	4.79 (1.19)	0.050	4.37 (0.73)	4.83 (1.06)	0.712
LDL-C ≥ 130, percentage (SE)	1.42 (0.46)	3.31 (1.07)	0.056	3.53 (0.67)	3.44 (0.96)	0.944
HDL-C < 40, percentage (SE)	17.08 (1.36)	12.17 (1.93)	0.049	21.68 (1.36)	14.30 (1.92)	0.003
TG ≥ 130, percentage (SE)	9.15 (1.03)	11.15 (1.99)	0.333	14.70 (1.21)	13.21 (2.06)	0.480
non-HDL-C ≥ 145, percentage (SE)	2.70 (0.61)	4.22 (1.08)	0.192	6.07 (0.83)	4.65 (1.00)	0.290
Physical activity (h/week), mean (SE)	4.60 (0.32)	6.67 (0.75)	0.011	4.85 (0.30)	6.84 (0.64)	0.005
**Female (*n* = 1488)**	**Normal (*n* = 1223)**			**Overweight or Obesity (*n* = 142)**		
	**Vitamin D deficiency (*n* = 993)**	**Vitamin D sufficiency (*n* = 230)**	***p***	**Vitamin D deficiency (*n* = 113)**	**Vitamin D sufficiency (*n* = 29)**	***p***
TC ≥ 200, percentage (SE)	8.04 (1.01)	6.62 (1.73)	0.505	8.84 (0.97)	6.89 (1.66)	0.346
LDL-C ≥ 130, percentage (SE)	6.11 (0.89)	5.09 (1.45)	0.572	6.93 (0.86)	6.16 (1.54)	0.676
HDL-C < 40, percentage (SE)	5.81 (0.89)	13.46 (2.74)	0.001	9.45 (1.05)	16.77 (2.70)	0.003
TG ≥ 130, percentage (SE)	8.29 (1.05)	12.30 (2.55)	0.102	10.86 (1.08)	13.84 (2.38)	0.222
non-HDL-C ≥ 145, percentage (SE)	6.60 (0.92)	6.84 (1.74)	0.904	8.20 (0.91)	8.36 (1.86)	0.937
Physical activity (h/week), mean (SE)	2.31 (0.21)	3.04 (0.60)	0.256	2.43 (0.19)	3.55 (0.57)	0.055

Values are presented as means (standard error), and categorical data are presented as percentages (standard error). Vitamin D deficiency and sufficiency was defined as 25OHD ≤ 20 ng/mL and > 20 ng/mL, respectively. BMI: body mass index; 25OHD: 25-hydroxyvitamin D; TC: total cholesterol; SE: standard error; LDL-C: low-density lipoprotein cholesterol; HDL-C: high-density lipoprotein cholesterol; TG: triglycerides.

**Table 3:** Proportion of subjects with vitamin D deficiency and physical activity according to BMI and lipid levels.

			TC < 200	TC ≥ 200	*p*	LDL-C < 130	LDL-C ≥ 130	*p*	HDL-C ≥ 40	HDL-C < 40	*p*	TG < 130	TG ≥ 130	*p*	non-HDL-C < 145	non-HDL-C ≥ 145	*p*
Male (*n* = 1695)	Normal (*n* = 1358)	No. of Participants (*n*)	1314	44		1331	27		1140	218		1224	134		1314	44	
		Vitamin D deficiency, percentage (SE)	75.80 (1.60)	60.39 (8.65)	0.050	75.70 (1.58)	56.65 (11.32)	0.056	74.25 (1.73)	81.09 (2.90)	0.049	75.75 (1.63)	71.48 (4.44)	0.333	75.63 (1.61)	66.16 (7.69)	0.192
		Physical activity (h/week), mean (SE)	5.13 (0.31)	4.55 (1.03)	0.589	5.15 (0.31)	3.32 (0.93)	0.060	5.26 (0.33)	4.31 (0.68)	0.192	5.11 (0.30)	5.17 (1.15)	0.954	5.13 (0.31)	4.59 (1.04)	0.619
	Overweight or obesity (*n* = 337)	No. of Participants (*n*)	304	34		305	32		218	119		237	100		334	53	
		Vitamin D deficiency, percentage (SE)	76.25 (1.44)	74.26 (5.37)	0.712	76.14 (1.43)	76.58 (6.03)	0.944	74.43 (1.64)	83.12 (2.23)	0.003	75.79 (1.51)	78.36 (3.29)	0.480	75.89 (1.46)	80.62 (3.98)	0.290
		Physical activity (h/week), mean (SE)	5.38 (0.28)	4.28 (0.71)	0.150	5.38 (0.28)	3.76 (0.69)	0.026	5.41 (0.29)	5.01 (0.70)	0.597	5.44 (0.29)	4.65 (0.67)	0.266	5.41 (0.29)	4.00 (0.58)	0.028
Female (*n* = 1488)	Normal (*n* = 1223)	No. of Participants (*n*)	1126	97		1151	72		1128	95		1107	116		1137	86	
		Vitamin D deficiency, percentage (SE)	82.28 (1.51)	85.14 (3.78)	0.505	82.35 (1.49)	84.98 (4.20)	0.572	83.69 (1.44)	67.04 (6.02)	0.001	83.14 (1.48)	76.08 (4.60)	0.102	82.54 (1.48)	81.99 (4.45)	0.904
		Physical activity (h/week), mean (SE)	2.37 (0.22)	3.33 (0.63)	0.150	2.37 (0.21)	3.58 (0.79)	0.137	2.46 (0.22)	2.22 (0.49)	0.661	2.50 (0.22)	1.86 (0.25)	0.059	2.39 (0.21)	3.08 0.67)	0.330
	Overweight or obesity (*n* = 265)	No. of Participants (*n*)	237	28		237	28		196	69		207	58		223	42	
		Vitamin D deficiency, percentage (SE)	82.77 (1.40)	86.31 (3.23)	0.346	82.96 (1.38)	84.66 (3.73)	0.676	84.23 (1.35)	73.45 (3.98)	0.003	83.55 (1.36)	79.39 (3.52)	0.222	83.10 (1.38)	82.80 (3.65)	0.937
		Physical activity (h/week), mean (SE)	2.55 (0.20)	3.41 (0.56)	0.146	2.56 (0.20)	3.47 (0.63)	0.175	2.62 (0.21)	2.60 (0.41)	0.956	2.69 (0.21)	2.04 (0.26)	0.056	2.57 (0.20)	3.13 (0.50)	0.307

Values are presented as mean (standard error) and categorical data as percentages (standard error). Vitamin D deficiency is defined as 25OHD ≤ 20 ng/mL. 25OHD: 25-hydroxyvitamin D; BMI: body mass index; TC: total cholesterol; LDL-C: low-density lipoprotein cholesterol; HDL-C: high-density lipoprotein cholesterol; TG: triglycerides; SE: standard error.
